# IFNλ4 Gene Variants Are Associated with CD8^+^ T Cell IL-2 Response After COVID-19 Vaccination: A Preliminary Study

**DOI:** 10.3390/biom16081111

**Published:** 2026-07-29

**Authors:** Giuseppa Luisa Sanfilippo, Giusto Davide Badami, Marco Pio La Manna, Mariangela Pizzo, Domenico Lio, Giovanni Maurizio Giammanco, Nadia Caccamo

**Affiliations:** 1 G. Giglio Institute Foundation, 90015 Cefalù, Italy; giuseppa.sanfilippo@hsrgiglio.it; 2 Central Laboratory of Advanced Diagnosis and Biomedical Research (CLADIBIOR), Azienda Ospedaliera Universitaria Policlinico (AOUP) Paolo Giaccone, University of Palermo, 90127 Palermo, Italy; giustodavide.badami@unipa.it (G.D.B.); marcopio.lamanna@unipa.it (M.P.L.M.); nadia.caccamo@unipa.it (N.C.); 3 Department of Biomedicine, Neurosciences and Advanced Diagnostic (Bi.N.D.), University of Palermo, 90127 Palermo, Italy; 4 Microbiology, Department of Health Promotion, Mother and Child Care, Internal Medicine and Medical Specialties (PROMISE), University of Palermo, 90127 Palermo, Italy; mpizzo@ismett.edu (M.P.); giovanni.giammanco@unipa.it (G.M.G.); 5 IRCCS ISMETT, 90127 Palermo, Italy; 6 University Research Center “Migrate”, University of Palermo, 90133 Palermo, Italy

**Keywords:** SARS-CoV-2 vaccine, IFN-lambda, single-nucleotide polymorphism (SNP), T-cell response, cytokine production

## Abstract

Individual responses to SARS-CoV-2 vaccination vary due to viral mutations, demographic factors, and genetic background, with T-cell responses being less affected by viral variability. However, less is known about the effect of age, sex, and genetic background on the T-cell response in vaccinated subjects. In particular, the influence of interferon lambda (IFN-λ) genetic polymorphisms on vaccine-induced cellular immunity has been poorly explored. Here, we assessed intracellular production of IFNγ, TNFα, and IL-2 in CD4^+^ and CD8^+^ T cells stimulated with SARS-CoV-2 spike peptides from 50 vaccinated subjects genotyped for *IFNL3* (*rs12980275* and *rs8099917*) and *IFNL4* (*rs11322783TT* and *rs12979860*) SNPs. We found that the *IFNL4 rs11322783TT/TT* genotype is associated with increased IL-2 production in CD8^+^ T cells, whereas the *ΔG* allele correlates with a reduced IL-2 response. No significant effects on CD4^+^ T-cell cytokine production or other cytokines were observed. These preliminary findings suggest that type III interferon gene variants might be involved in CD8^+^ T-cell responses post-vaccination, opening the possibility that evaluation of *IFNL4* SNPs might be useful in designing strategies of personalized vaccination.

## 1. Introduction

Vaccines have played a remarkable role in the management and containment of the SARS-CoV-2 pandemic, significantly contributing to reducing the severity of infections [1]. The most widely used formulations contain a modified messenger RNA sequence encoding the virus’s Spike protein [2]. These vaccine formulations induce both antibody and cellular immune responses, with significant involvement of T helper 1 (Th1) and T helper 2 (Th2) and T cytotoxic pathways [3]. On the other hand, a significant component of the antiviral response is the production of IFN-I and IFN-III, in particular against SARS-CoV-infection [4]. IFN-I and IFN-III production is induced by pathogen-associated molecular pattern (PAMP) or damage-associated molecular pattern (DAMP) interaction with pattern recognition receptors, such as TLR molecules (PRRs) [5], expressed in particular on mucosal epithelia [6]. SARS-CoV-2 infection induces IFN-I and IFN-III in different in vitro models: human airway epithelial (HAE) cells, nasal epithelial cells, and immortalized cell lines [7,8]. In vivo, interaction between SARS-CoV-2 and TLR7 receptors activates plasmacytoid dendritic cells (pDCs) in the lung with the production of IFN-α and IFN-λ1, which leads to activation of lung macrophages [9,10]. The same results have been obtained using peripheral blood mononuclear cells as an in vitro model for the immune-cell response to SARS-CoV-2 [10].

So far, four types of interferon III have been identified (IFN-λ1-4) [11]. Unlike type I interferons (such as IFN-α and IFN-β), IFN-λ exerts an antiviral action, mainly localized in the epithelial cells of the mucosal tract, limiting viral infection and systemic inflammation [12]. The central molecular mechanisms of immune-cell activation by IFN-λ are the activation of the Janus kinase (JAK) signal transducer and activator of transcription (STAT) pathway by initiating the phosphorylation of JAK1 and tyrosine kinase 2 (TYK2), which subsequently phosphorylate STAT1 and STAT2. Phosphorylated STAT heterodimers binding to cytoplasmic IRF9 form the IFN-stimulated gene factor 3 (ISGF3) complex and, in the nucleus, activate the interferon-stimulated response elements (ISREs) involved in polarization of macrophages and dendritic cells, activation and proliferation of T cells, and, in turn, cytokine gene stimulation [13].

The *IFNL3* and *IFNL4* genes, encoding, respectively, IFN-λ3 and IFN-λ4, have been widely studied in the past for their role in the clearance of HCV (hepatitis C) virus, but in recent years, attention has been focused on their potential modulatory role in COVID-19 [14,15,16,17,18,19].

In addition, data obtained by different research groups indicate a correlation between IFN-λ levels and COVID-19 severity, with worse effects in low-producing subjects [20,21].

The intensity and effectiveness of the interferon response are influenced by both viral and host factors, with the host’s genetic background playing a significant role [20,21]. Several studies have shown that certain single-nucleotide polymorphisms (SNPs) in type III interferon genes can affect the immune response to SARS-CoV-2 and the clinical outcomes of COVID-19. Among these, rs8099917 and rs12979860 are the most extensively studied, as they influence the expression levels of IFN-λ3 and IFN-λ4, respectively [15,16,17,18,19]. Recent research indicates that individuals carrying the favorable C allele of rs12979860 tend to mount a more effective antiviral response and are at lower risk of severe COVID-19, likely due to enhanced induction of interferon-stimulated genes (ISGs) in the upper airways [17]. In contrast, the TT genotype is associated with a higher risk of severe or fatal COVID-19, possibly due to a weaker and delayed IFN-λ response.

Strict genetic control affects IFN-λ4 production. *rs11322783 ΔG/TT* is characterized by the presence of a G deletion (ancestral *ΔG* allele) or of an insertion of a *TT* frameshift. The presence of the *ΔG* allele is associated with the transcription and synthesis of a functional IFN-λ4 molecule, whereas the presence of the homozygote *TT/TT* genotype blocks the synthesis of IFN-λ4 [22]. The presence of the *IFNL4 rs11322783TT/TT* SNPs seems to be associated with a better prognosis of COVID-19 [15,16].

However, less is known about the role that genetic variants of type III interferons (IFN-λ) may play in modulating the efficacy of the immune response induced by SARS-CoV-2 vaccination. In a recent study, we reported that cytokine gene polymorphisms, which are key regulators of inflammation and antibody production, can influence the magnitude of the antibody response to vaccination [23]. Therefore, in this study, we evaluated whether genetic variants in IFN-λ3 and IFN-λ4 are associated with differences in the cellular immune response—specifically, the in vitro response of CD4^+^ and CD8^+^ T cells to SARS-CoV-2 spike protein peptides—in vaccinated individuals.

## 2. Materials and Methods

### 2.1. Subjects

Peripheral blood samples were obtained from 50 healthy subjects (25 males and 25 females, with mean age of 52 years) that received a complete cycle (three or four doses) of the Pfizer/BioNTech BNT162b2 mRNA vaccine. This study was performed according to the Declaration of Helsinki and was approved by the Ethical Committee of A.O.U.P. “P. Giaccone” Hospital (protocol code 09/2021). All participants gave their informed consent, and data were encoded to ensure privacy protection of the subjects.

### 2.2. Isolation and Culture of Peripheral Blood Mononuclear Cells (PBMCs)

Peripheral blood mononuclear cells (PBMCs) were isolated from blood samples using a Ficoll-Paque density gradient (Lympholyte, Cederlane, Burlington, ON, Canada) centrifugation method and promptly stored in liquid nitrogen until use. PBMCs were thawed, resuspended in complete RPMI 1640 medium supplemented with 10% FBS (all from Sigma-Aldrich, Saint Louis, MO, USA) and washed twice. The PBMC viability (>95%) was controlled using Trypan blue staining.

PBMCs were cultured with TexMACS™ (Miltenyi Biotec, Bergisch Gladbach, Germany) or RPMI-1640 medium + 5% human serum + BSA/FBS) in 24-well plates at a density of ~400,000 cells/mL. Each sample was cultured in triplicate and stimulated with peptides (Table 1) of the SARS-CoV-2 spike protein (PepTivator^®^ SARS-CoV-2 Prot_S,S1,S+ (Miltenyi Biotec) at a concentration of 0.6 nmol ≈ 1 µg/mL). Negative (containing unstimulated cells) and positive (cells stimulated with CytoStim™ Miltenyi Biotec, a polyclonal TCR/MHC activator at a concentration of 200 µL per ~10^8^ cells) controls were set up for each sample. After 1 h at 37 °C with 5% CO_2_, 10 µg/mL monensin (BioLegend, Amsterdam, The Netherlands) was added as a Golgi blocker, and plates were incubated for 18–24 h at 3 °C with 5% CO_2_.

### 2.3. Flow Cytometry of Peptide-Stimulated Cells

After incubation, cells were washed with PBS and stained with the Zombie NIR Fixable Viability kit (BioLegend 423106) to assess cell viability. Cultured cell samples were incubated with anti-CD3, -CD4, and -CD8 REAfinity™ monoclonal antibodies (Miltenyi Biotec); fixed and permeabilized (Inside Stain Kit, Miltenyi Biotec); and subsequently incubated with moAbs specific for TNFα, IFNγ, and IL-2 (Miltenyi Biotec) [24]. Staining was performed using monoclonal antibodies (Miltenyi Biotec), which are recombinant antibodies specifically engineered to eliminate Fc receptor-mediated binding. Consequently, these antibodies do not interact with Fcγ receptors expressed on monocytes, B cells, NK cells, or other immune-cell subsets, thereby minimizing non-specific staining and ensuring high binding specificity.

Flow cytometric acquisition of labeled PBMCs was performed using the FACS Lyric flow cytometer (BD Biosciences, San Jose, CA, USA), acquiring a minimum of 200,000 total events for each sample in the lymphocyte gate (based on FSC/SSC). Finally, analysis was performed with FlowJo software v10 (Treestar Inc., Ashland, OR, USA) as previously described [24]. Briefly, gating follows the strategy Live cells →Single events → CD3^+^ → CD4^+^/CD8^+^ → cytokine^+^ subsets (Figure S1), ensuring reproducibility of the data. A minimum of 50,000 CD3^+^ cells per sample was required as a quality control (QC) threshold. Results were expressed as the percentage of cytokine-positive CD4^+^ and CD8^+^ cells (TNFα^+^, IFNγ^+^, or IL-2^+^) compared with total CD4^+^ and CD8^+^ T cells.

### 2.4. Cytokine SNP Typing

Blood samples, in tripotassium EDTA sterile tubes, were used for DNA extraction (“Magna Pure” 24 System automated extraction method; Roche Diagnostics S.p.A., Monza (MB), Italy). As reported in Table 2, we selected seven functional and common SNPs of the *IFNG*, *TNFA*, *IL2*, *IFNL3* and *IFNL4* genes (http://www.ensembl.org/index.html, last accessed on 18 December 2025). DNA samples were typed as previously described [23] by using the KASPar assay (K-Bioscience Ltd. Hoddesdon, UK). Genotypes analysis was performed on 7300 system SDS software, version 1.3 (Applera Italia, Monza (MI), Italy), as previously described.

### 2.5. Statistics

Mean ± standard deviation and the median of the continuous variables (age, percentage of cells positive for cytoplasmic cytokines and percentage of the increase in intra-cytoplasmic cytokine production) were calculated, and modal distribution was verified by the Kolmogorov and Smirnov method and analysis of variance. When data did not follow a normal distribution model, the non-parametric Mann–Whitney test was applied.

To analyze the data, we stratified the subjects into two subsets: subjects that had a percentage of increase in intra-cytoplasmic cytokine production under the median values and subjects with a percentage of increase in cytokine production equal to or greater than the median. We analyzed contingency tables containing the following categories: subjects positive for a genotype and with a cytokine production increase percentage < median (A); genotype-negative and increase <median (B); positive for a genotype and cytokine production ≥ median (C); negative for a genotype and cytokine production ≥ median (D).

The minimum calculation power was evaluated using an online tool (https://clincalc.com/stats/samplesize.aspx, accessed on 3 July 2026).

Genotype and allele frequencies of the different gene polymorphisms were assessed based on gene counts. Goodness of fit between observed and expected genotype frequencies (Hardy–Weinberg equilibrium) of the population recruited was preliminarily analyzed using Pearson distribution and χ2 tests. Significant differences in allele and genotype distributions between the subgroups were calculated using Fisher’s exact test. Multiple logistic regression models were also applied using dominant (major allele homozygotes versus heterozygotes plus minor allele homozygotes) and recessive (major allele homozygotes plus heterozygotes versus minor allele homozygotes) models. Odds ratios (ORs), 95% confidence intervals (95% CIs), and *p*-values (*p*-value cutoff < 0.05) were determined using GraphPad InStat software version 3.06 (GraphPad, San Diego, CA, USA) and an online SNP-devoted statistical analysis tool (https://www.snpstats.net/start.htm, last accessed on 24 January 2026). As multiple comparisons were made, we tested the sequentially rejective hypothesis using an online tool (https://r-statistics.co/tools/multiple-testing-correction.html, last accessed on 24 July 2026) that adjusts significant *p*-values by applying Bonferroni, Holm, Hochberg and Hommel corrections. When appropriate, the Mantel–Haenszel procedure for the trend analysis was applied.

## 3. Results

Table 3 shows the median (95% confidence interval, CI) of percentage and increase in CD4^+^ and CD8^+^ cells positive for intra-cytoplasmic cytokines (IFNγ, TNFα, and IL-2) after stimulation with a mix of synthetic peptides homologous to the virus spike protein. A minimum calculation power of 75% was found for evaluating high-responder (percentage of increase in cytokine production ≥ median) vs. low-responder (percentage of increase in cytokine production < median) samples. This level was retained for major allele and genotype analyses (see below).

We observed that the CD4^+^ population had increases of approximately 10% in cells positive for IFNγ or TNFα and of 13% in cells positive for IL-2. Analysis of the response of CD8^+^ cells indicated a similar increase in the percentage of IFNγ^+^ cells, while more significant increases were observed in TNFα^+^ cells (31%) and IL-2 (32.5%).

Since age and gender are factors that might influence vaccine protection against SARS-CoV-2 infection [29], we evaluated whether these two variables influenced the CD4^+^ or CD8^+^ T-cell response to peptide stimulation. As reported in Table 4 and Table 5, neither age nor sex seemed associated with a different increase in cytokine (IFNγ, TNFα, and IL-2)-positive CD3^+^ CD4^+^ and CD3^+^ CD8^+^ cells after SARS-CoV-2 peptide stimulation.

As reported in a previous paper, the polymorphisms of some cytokine genes can influence the effectiveness of the response to SARS-CoV-2 vaccination [23]. We preliminarily evaluated whether polymorphisms capable of modifying the production of IFNγ, TNFα, and IL-2 could influence the response to synthetic peptides analogous to sequences of the SARS-CoV-2 spike protein. The results obtained demonstrated that the production of neither IFNγ, TNFα, nor IL-2 is influenced by the SNPs *INFG rs2830561A/T*, *TNFA rs1800629G/A*, and *IL-2 rs2069762A/C*, respectively, in CD4^+^ and CD8^+^ T lymphocytes subsets after in vitro stimulation with SARS-CoV-2 virus peptides (Supplementary Tables S1–S3).

Considering that type III interferons play a fundamental role in mucosal immunity against viral infections, in particular in the respiratory tract, including SARS-CoV-2 [11], we evaluated whether polymorphisms in the genes encoding IFN-λ3 and -4 could influence the response of CD4^+^ and CD8^+^ T lymphocytes from subjects vaccinated against the virus to in vitro stimulation with SARS-CoV-2 spike protein-analog peptides.

The results obtained indicate that the *IFNL3* and *IFNL4* SNPs analyzed did not influence the increase in intra-cytoplasmic levels of IFNγ and IL-2 in CD4^+^ cells stimulated with SARS-CoV-2 virus spike-analog peptides (Supplementary Tables S4 and S5).

The same results were obtained when evaluating the percentage increase in TNFα-producing CD3^+^ CD4^+^ cells. However, a roughly significant tendency for a reduced increase in TNFα^+^ cells was identified in carriers of the G/T genotype of *IFNL3* rs8099917 with respect to the homozygous genotypes (test for trend *p* = 0.05). No significant associations were observed for the *IFNL4 rs11322783* SNP (Table 6).

None of the analyzed variants showed a statistically significant effect on the increase in CD8^+^ cells producing IFNγ nor TNFα (Supplementary Tables S6 and S7).

On the other hand, the presence of the *TT/TT* genotype of *IFNL4 rs11322783* appears to be significantly associated with a greater increase in the percentage of IL-2-positive CD8^+^ cells, while the presence of the ΔG allele, both as heterozygous genotypes as well as in the dominant model (*ΔG/TT + ΔG/ΔG vs. TT/TT*), appeared to be associated with a reduced increase (Table 7). As a multiple comparison was made, an online tool (https://r-statistics.co/tools/multiple-testing-correction.html, last accessed on 24 July 2026) that adjusts significant *p* values by applying Bonferroni, Holm, Hochberg and Hommel corrections to test the rejective hypothesis was applied (Table S8). After correction (six genotypes of the two IFNL4 SNPs), association of the *IFNL4 ΔG/TT* genotype with IL-2 attained a significant *p*-value, whereas the *IFN-λ4 TT/TT* genotype did not.

Analysis of the *IFNL4 rs12979860* genotypes did not allow us to find any association, but a significant trend for a lower increase (a <median increase in 32% of samples vs. 8% of samples with a ≥median increase) in intra-cytoplasmic IL-2 production by CD8^+^ T lymphocytes stimulated with spike protein peptides was found for the *IFNL4 rs12979860C/C* genotype (test for trend *p* = 0.0385).

Taken together, these data open the possibility that specific polymorphisms in genes encoding type III interferons might be associated with differences in the increase in intra-cytoplasmic IL-2 production by CD8^+^ T lymphocytes in response to antigenic determinants of the SARS-CoV-2 spike protein in vaccinated subjects.

## 4. Discussion

The individual response to the SARS-CoV-2 vaccine and the ability to maintain effective protection over time appear to be influenced by several factors. In particular, viral variability, age, sex, and individual genetic background can influence the production of specific antibodies against the virus [23]. Change in virus immunogenic epitopes affect the efficacy of neutralizing antibodies, whereas the T-cell response appears better conserved [30,31]. However, less is known about the role of age, sex, and genetic background in influencing the T-cell response in subjects vaccinated against the SARS-CoV-2 virus. Therefore, we evaluated whether relevant factors regulating the antiviral response, such as age and sex, and some key cytokine polymorphisms involved in the regulation of the antiviral response [32] could play a role in modulating the cellular response to vaccines against SARS-CoV-2.

The adaptive immune T-cell response was evaluated, using flow cytometry to assess proinflammatory cytokine production (IFNγ TNFα, and IL-2) in CD4^+^ and CD8^+^ T cells stimulated with spike-related immunogen peptides. Peptide stimulation of CD4^+^ T cells induced a modest increase in intra-cytoplasmic cytokine production, whereas stimulation of CD8^+^ T cells induced a more significant increase in the production of cytokines, in particular of TNFα and IL-2. Our results are aligned with those of others and our research group indicating that anti-SARS-CoV-2 vaccination elicits in particular CD8^+^ T-cell responses [24].

Moreover, our findings showed that anti-spike T-cell reactivity is not influenced by age and sex. These results are in good agreement with evidence obtained in a longitudinal study by a large research consortium [33].

In order to evaluate the effects of polymorphisms of genes coding for the cytokines (IFNγ, TNFα, and IL-2) measured with the cytofluorimetric assay, we typed functional polymorphisms (*IFNG rs2430561 T/A*, *TNFA rs1800629 G/A*, and *IL2 rs2069762A/C*), known to be involved in modulation of cytokine production. The *rs2430561T/A* SNP of the *IFNG* gene is located within a putative NF-κB binding site. The A allele reduces the affinity for NF-κB transcription factor and, in turn, low levels of IFNγ production [26]. The *TNFA rs1800629A* allele is associated with higher levels of TNFα than the G allele, increasing the binding of a transcription factor to the promoter region of the *TNFA* gene [27]. Studies on the *rs2069762A/C* polymorphism of the *IL2* gene promoter region have demonstrated that *rs2069762CC* homozygous subjects are able to produce the highest amount of IL-2 [25]. In spite of these reports, we were unable to find a relationship between these functionally relevant SNPs and the percentage increase in CD4^+^ or CD8^+^ T cells positive for the related intra-cytoplasmic cytokines after stimulation with spike-related immunogen peptides.

The first line of intra- and extracellular defense against viral infection involves two types of “natural” interferons, type I IFN (IFN-α and -β) and type III IFN (IFN-λ), which “interfere” with the initial viral replication [34]. Type III IFNs are essential components of the mucosal innate immune response, with reported in vitro antiviral activity against respiratory infections, such as *Mycobacterium tuberculosis*, influenza A virus, respiratory syncytial virus, and coronaviruses (MERS-CoV and SARS-CoV-2) [35]. On the other hand, a significant bulk of data indicates that both type I IFNs and type III IFNs are impaired in patients with SARS-CoV-2 [36]. Moreover, subjects expressing low levels of IFN-λ are at significant risk of developing severe COVID-19 [20], even though contrasting results have been published [37].

At this moment, four IFN-λ subtypes are known in humans: IFN-λ1 (also known as IL-29), IFN-λ2 (IL-28A), IFN-λ3 (IL-28B), and IFN-λ4. The amino acid sequence of IFN-λ4 differs substantially (sharing only ~28% amino acid identity) from the other IFN-λ genes [16]. In addition, only about 50% of Caucasian individuals produce a functional IFN-λ4 protein [16]. Actually, the presence of the ancestral *IFNL4-ΔG* allele of the *Ins/Del rs11322783* polymorphism enables synthesis of the full-length functional IFN-λ4 protein. On the contrary, the *IFNL4 rs11322783TT* genotype introduces a frameshift in the open reading frame, which blocks the production of IFN-λ4 protein [22].

Many studies have shown that the presence of some polymorphisms of the *IFNL3* and *IFNL4* genes can influence the severity of SARS-CoV-2 infection [15,16,17,38,39]. The major concordant findings of these research studies indicate that the presence of “favorable” genotypes of *IFNL3* (*rs12980275AA* and *rs8099917TT*) and/or *IFNL4* SNPs (*rs11322783TT/TT* and *rs12979860CC*) was associated with a better prognosis of COVID-19, favoring rapid clearance of SARS-CoV-2 virus particles [15,16,17]. In addition, Nardy et al. [18] have recently reported that the presence of the *IFNL4* rs12979860CC genotype is associated with an increased titer of anti-SARS-CoV2 IgG in vaccinated subjects.

Starting from this well-established framework, we evaluated whether the presence of these polymorphisms could also influence the in vitro response of T lymphocytes from vaccinated subjects to stimulation with spike protein peptides. Our results indicate that none of the *IFNL3* and *IFNL4* SNP genotypes is associated with differences in the level of increase in intra-cytoplasmic levels of IFNγ, TNFα and IL-2 in CD4^+^ T cells stimulated with SARS-CoV-2 virus spike-analog peptides. Our findings seem to suggest that the activation of circulating antigen-specific CD4^+^ T cells with a Th1 intra-cytoplasmic cytokine profile is not influenced by the *IFNL3* or *IFNL4* genetic variants. Similar results were obtained when evaluating the percentage increase in intra-cytoplasmic IFNγ and TNFα in antigen-specific CD8^+^ T lymphocytes.

On the other hand, analysis of the association of *IFNL4* SNPs with the increase in intra-cytoplasmic IL-2 in antigen-stimulated CD8^+^ T cells allowed us to hypothesize that the positivity for the *TT/TT* genotype of *IFNL4 rs11322783*, even if not significant after correction, might be associated with a greater increase in IL-2. On the other hand, the presence of the *ΔG* allele in the heterozygous genotype appears statistically associated with a reduced level of intra-cytoplasmic IL-2 production. We cannot demonstrate a significant association of the ΔG/ΔG homozygous genotype with a reduced production of IL-2 due to the low number of subjects positive for the genotype.

As reported above, IFN-λ4 production is conditioned by the presence of the ancestral *ΔG* allele, whereas the insertion of a *TT* frameshift blocks the synthesis of this IFNIII subtype [27]. So, our data might suggest that a genetically determined lack of IFN-λ4 might be involved in the observed decreased production of intra-cytoplasmic IL-2 in CD8^+^ T cells stimulated in vitro with spike-analog peptides. This result appears counterintuitive, considering the role of IFN-λ in inhibiting and containing viral proliferation in the early stages of SARS-CoV2 infection [34]. On the other hand, a large number of data seem to indicate that the presence of the *rs11322783ΔG* allele and therefore the production of IFN-λ4 have negative effects on viral clearance [15,38,40], on the immune response [17,21] and on the clinical severity of patients affected by COVID-19 [17,41,42]. Data consistently reported in the literature confirm the association of the *rs11322783ΔG* allele with reduced capacity to clear other respiratory viruses [43], as well as with the reduced capacity to clear other viruses. As is well known, the first reports regard the HCV virus and the reduced viral clearance and response to therapy associated with the *rs11322783ΔG* allele [22,44,45]. Less is known on the impact of this polymorphism on the response to a vaccination. As an example, the *∆G* allele was associated with a reduced probability to develop protective anti-HBs titers in response to HBV vaccination or infection [46].

Although our findings require confirmation in a larger cohort of vaccinated subjects, they are consistent with the broader literature [15,16,38,40,41,42]. However, functional experiments, such as stimulation in presence of recombinant IFN-λ4 of PBMCs with different genotypes, are necessary to explore the possible biological mechanisms involved in reduced IL-2 production by CD8^+^ T cells from *IFNL4 rs11322783ΔG-*positive subjects. The current knowledge on IFN-λ biological effects indicates that these mechanisms are mediated by the activation of the JAK/STAT pathway, which, in turn, through IFN-stimulated gene factor 3, activates interferon-stimulated response elements (ISREs) [47]. Activation of ISREs induces a large array of cellular effects, such as polarization of macrophages and dendritic cells and, in turn, activation and proliferation of T cells [13]. In an in vitro study, using liver cell lines, Chen et al. [47] demonstrated that functional IFN-λ4 stimulates potent JAK-STAT signaling, but IFN-λ4 expression inhibits HCV peptide-specific CD8^+^ T-cell activation, whereas the presence of the *IFNL4 rs11322783TT* non-functional genotype does not interfere with CD8^+^ T-cell activation. In addition, functionally active IFN-λ4 reduces the effects of long-term inflammation. Liver chronic inflammation due to viral infections or nonalcoholic fatty liver disease is attenuated by functionally active IFNλ4 [48].

Recent studies indicate that a role might be played by the suppressors of cytokine signaling (SOCS) protein family. Excessive JAK-STAT signaling may activate SOCSs, which, in turn, reduce IFN-λ stimulatory effects [49]. SOCS proteins play a crucial role in maintaining the immune balance by providing negative feedback regulation of cytokine signaling, thereby preventing excessive immune responses that could harm the host during infections [50]. In particular, SOCS1 shows selective regulation of IFN, IL-12 and IL-2 family signaling [49]. Considering this overall picture, a plausible hypothesis might be that in *IFNL4 rs11322783 ΔG* allele-positive subjects, the production of IFNλ4 induced by SARS-CoV2 infection or after vaccination might stimulate the SOCS pathway and attenuate the immune response. This might induce the activation of a relatively reduced number of antigen-specific CD8^+^ cells and, in turn, in our experimental conditions, a reduced proportion of CD8 ^+^ IL-2^+^ T cells after in vitro stimulation.

Regarding *IFNL*4 *rs12979860*, an association of *T*-positive genotypes with severe COVID-19 has been reported by different research groups [15,38,51,52], whereas some studies report a protective role for the *TT* genotype [42]. Our results show that the *IFNL*4 *rs12979860* genotypes were not related to an increased level of intra-cytoplasmic cytokines after spike peptide stimulation. However, we found a significant trend indicating that reduced IL-2 intra-cytoplasmic production might be associated with the *IFNL*4 *rs12979860CC* genotype. Considering that previous studies showed a sustained inflammatory response in patients with infection bearing the *T* allele [17], we might hypothesize that relatively low inflammatory stimulation in vaccinated subjects might lead to reduced CD8^+^ recruitment and anti-SARS-CoV-2 priming. In this view, it is intriguing that Hayashi et al. [53] have recently reported that a complete cycle of anti-SARS-CoV-2 vaccination may reduce the production of IFN-λ, in particular IFN-λ3. So, it is possible that vaccination might modulate the effect of IFN-λ on the type and intensity of cellular immune responses, impinging on vaccine effectiveness.

## 5. Limitations

This preliminary study had some limitations that should be taken into account. The most significant limitation of this study is the small cohort of 50 participants, which makes this study underpowered for genetic association analyses. In consequence, several genotype subgroups contained very few individuals, and the statistical results corrected for multiple comparisons do not confirm some associations. Moreover, even if the evaluation of calculation power shows that 75% of calculation power was found for the major allele-positive genotype frequencies of the selected SNP comparisons, a minor level of calculation power (25–45%) was found when the minor allele homozygotes were analyzed. So, our results should be treated with caution considering the impact of the small sample size on false-negative rates and the precision of effect-size estimation.

## 6. Conclusions

In conclusion, our data, although requiring confirmation in a larger population, represent preliminary evidence that specific type III interferon gene variants may be associated with individual variability in the immune response to COVID-19 vaccines. In particular, genetically controlled IFN-λ4 production may represent an interesting target for further research, considering, in perspective, the possibility to design personalized vaccination strategies.

## Figures and Tables

**Table 1:** Peptide pools used for stimulation.

Peptide Mix	Protein Region	Peptide Pool Characteristics
Prot S	Whole spike protein (AA 5-1273)	15-mer peptide pool (overlap of 11 aa)
Prot S1	S1 spike protein subunit	15-mer peptide pool (overlap of 11 aa)
Prot S+	Whole spike protein	15-mer peptide pool (overlap of 11 aa) (standardized for 1 × 10^6^ cell stimulation)

**Table 2:** SNPs typed.

Gene	SNP	Position	Major Allele	Minor Allele	Biological Effect	Ref.
*IL2*	*rs2069762*	4:122456825	A	C	The C allele is related to increased expression of IL-2	[25]
*IFNG*	*rs2430561*	12:68158742	A	T	The minor allele is associated with greater production of the cytokine	[26]
*TNFA*	*rs1800629*	6:31575254	G	A	The minor allele is associated with greater production of the cytokine	[27]
*IFNL3*	*rs12980275*	19:39241143	A	G	The A allele is related to increased production of IFN-λ3	[19]
	*rs8099917*	19:39252525	T	G	The T allele is related to increased expression of IFN-λ3	[28]
*IFNL4*	*rs11322783*	19:39248514	ΔG	TT	The TT allele inserts a frameshift that abolishes INFλ4 synthesis	[22]
	*rs12979860*	19:39248147	C	T	The C allele is related to increased expression of interferon λ4	[28]

**Table 3:** Evaluation of the median (95% CI) increase in percentage of CD4^+^ and CD8^+^ T cells positive for intra-cytoplasmic cytokines (IFNγ, TNFα, and IL-2) after stimulation with synthetic peptides homologous to different SARS-CoV-2 virus variants of the “spike” glycoprotein of T cells from 50 vaccinated subjects.

T-Cell Populations		IFNγ^+^	TNFα^+^	IL-2^+^
CD4^+^	Nr	50	50	50
	Neg Control	0.00 (0.007–0.10)	1.03 (0.78–1.61)	0.00 (0.01–0.02)
	Peptide Mix	0.01 (0.01–0.04)	0.88 (0.66–1.58)	0.02 (0.01–0.03)
	% Increase	11.0 (4.39–17.61)	1.0 (0.56–52.30)	11.0 (8.12–18.28)
CD8^+^	Nr	50	50	50
	Neg Control	0.00 (0.003–0.09)	0.09 (0.015–0.50)	0.001 (0.0019–0.060)
	Peptide Mix	0.01 (0.013–0.06)	0.13 (0.07–0.53)	0.035 (0.010–0.27)
	% Increase	9.00 (4.84–22.92)	31.0 (16.2–87.2)	32.5 (19.61–316.4)
	*p* *	0.776	0.0258	0.0025

* Evaluation of significant differences for CD4^+^ intra-cytoplasmic cytokine increase vs. CD8^+^ intra-cytoplasmic cytokine increase (Mann–Whitney non-parametric test).

**Table 4:** Effect of age ^1^ on the increase in percentage of CD4^+^ and CD8^+^ T cells positive for intra-cytoplasmic IFNγ, TNFα, or IL-2 (<median or ≥median) after stimulation with SARS-CoV-2 virus peptides of peripheral blood T cells from 50 vaccinated subjects.

	Intra-Cytoplasmic Cytokine Increase		Age			OR (95% CI)	*p*
			Nr.	<Median Age	≥Median Age		
				27	23		
CD4^+^	IFNγ^+^	<Median	Nr. (%)	12 (44.4)	16 (69.6)	0.35 (0.11–1.13)	0.093
		≥Median		15 (55.6)	7 (30.4)		
	TNFα^+^	<Median	Nr. (%)	16 (59.3)	11 (47.8)	1.59 (0.52–4.87)	0.570
		≥Median		11 (40.7)	12 (52.2)		
	IL-2^+^	<Median	Nr. (%)	14 (51.9)	8 (34.8)	2.02 (0.64–6.33)	0.264
		≥Median		13 (48.1)	15 (65.2)		
CD8^+^	IFNγ^+^	<Median	Nr. (%)	17 (63)	15 (65.2)	0.91 (0.28–2.89)	1.000
		≥Median		10 (27)	8 (34.8)		
	TNFα^+^	<Median	Nr. (%)	13 (48.1)	12 (52.2)	0.85 (0.29–2.59)	1.000
		≥Median		14 (51.9)	11 (47.8)		
	IL-2^+^	<Median	Nr. (%)	13 (48.1)	12 (52.2)	0.85 (0.28–2.59)	1.000
		≥Median		14 (51.9)	11 (47.8)		

^1^ Age mean ± S.D: 50.8 ± 14.2; median (95% CI): 53.5 (46.7–54.8).

**Table 5:** Effect of gender (50% female) on the increase (<median or ≥median) in percentage of CD4^+^ and CD8^+^ T cells positive for intra-cytoplasmic cytokines (IFNγ, TNFα, and IL-2) after stimulation with SARS-CoV-2 virus peptides of T cells from 50 vaccinated subjects.

Intra-Cytoplasmic Cytokine Increase				Males	Females	OR (95% CI)	*p*
CD4^+^	IFNγ^+^	<Median	Nr. (%)	14 (56)	14 (56)	1.00 (0.33–3.06)	1.000
		≥Median		11 (44)	11 (44)		
	TNFα^+^	<Median	Nr. (%)	14 (56)	13 (52)	1.17 (0.39–3.58)	1.000
		≥Median		11 (44)	12 (48)		
	IL-2^+^	<Median	Nr. (%)	8 (32)	14 (56)	0.37 (0.17–1.17)	0.154
		≥Median		17 (68)	11 (44)		
CD8^+^	IFNγ^+^	<Median	Nr. (%)	18 (72)	14 (56)	2.02 (0.62–6.56)	0.377
		≥Median		7 (28)	11 (44)		
	TNFα^+^	<Median	Nr. (%)	12 (48)	13 (52)	0.85 (0.28–2.59)	1.000
		≥Median		13 (52)	12 (48)		
	IL-2^+^	<Median	Nr. (%)	11 (44)	14 (56)	0.62 (020–1.89)	0.572
		≥Median		14 (56)	11 (44)		

**Table 6:** Association of *IFNL*3 and *IFNL*4 SNP genotypes with the increase (<median or ≥median) in TNFα-positive CD4^+^ T-cell percentage after stimulation with SARS-CoV-2 virus peptides.

SNP	Genotype	<Median Nr. (%)	≥Median Nr. (%)	OR (95% CI)	*p*
		27	23		
*IFNL3 rs12980275A/G*	A/A	6 (22.2)	3 (13.0)	1.91 (0.42–8.67)	0.865
	A/G	18 (66.7)	16 (69.6)	0.87 (0.27–2.89)	1.000
	G/G	3 (11.1)	4 (17.4)	0.59 (0.13–2.98)	0.687
*IFNL3 rs8099917G/T*	T/T	4 (14.8)	3 (13)	1.16 (0.23–5.82)	1.000
	G/T	23 (85.2)	14 (60.9)	3.70 (0.96–14.3)	0.062 ^a^
	G/G	0	6 (26.1)	---	---
*IFNL4 rs11322783ΔG/TT*	TT/TT	17 (63)	12 (52.2)	1.56 (0.50–4.83)	0.568
	ΔG/TT	9 (33.3)	9 (39.1)	0.78 (0.24–2.48)	0.771
	ΔG/ΔG	1 (0.7)	2 (8.7)	0.40 (0.03–4.77)	0.588
*IFNL4 rs12979860C/T*	C/C	5 (18.5)	5 (21.8)	0.82 (0.20–3.28)	1.000
	C/T	18 (66.7)	15 (65.2)	1.07 (0.33–3.45)	1.000
	T/T	4 (14.8)	3 (13.0)	1.16 (0.23–5.82)	1.000

^a^ Test for trend *p* = 0.05 (Mantel–Haenszel procedure).

**Table 7:** Association of *IFNL*3 and *IFNL*4 SNP genotypes with the increase (<median or ≥median) in IL-2^+^ CD8^+^ T-cell percentage after stimulation with SARS-CoV-2 virus peptides.

SNP	Genotype	<Median N (%)	≥Median N (%)	OR (95% CI)	*p*
		25	25		
*IFNL3 rs12980275A/G*	A/A	6 (24)	3 (12)	2.32 (0.51–10.6)	0.464
	A/G	16 (64)	18 (72)	0.69 (0.21–2.29)	0.762
	G/G	3 (12)	4 (16)	0.72 (0.14–3.59)	1.000
*IFNL3 rs8099917G/T*	T/T	6 (24)	2 (8)	3.63 (0.66–20.1)	0.247
	G/T	17 (68)	22 (88)	0.29 (0.07–1.26)	0.171
	G/G	2 (8)	1 (4)	2.09 (0.18–24.6)	1.000
*IFNL4 rs11322783ΔG/TT*	TT/TT	10 (40)	19 (76)	0.21 (0.06–0.71)	0.021
	ΔG/TT	14 (56)	4 (16)	6.68 (1.77–25.2)	0.007 ^a^
	ΔG/ΔG	1 (4)	2 (8)	0.48 (0.04–5.65)	1.000
*IFNL4 rs12979860C/T*	C/C	8 (32)	2 (8)	5.41 (1.02–28.8)	0.074 ^b^
	C/T	16 (64)	18 (72)	0.69 (0.21–2.29)	0.762
	T/T	1 (4)	5 (20)	0.17 (0.02–1.55)	0.189

^a^ Dominant model (ΔG/TT + ΔG/ΔG vs. TT/TT): OR (95%CI) = 4.75 (1.41–16.06) *p* = 0.021. ^b^ Test for trend *p* = 0.0385 (Mantel–Haenszel procedure).

## Data Availability

All data generated or analyzed during this study are stored in electronic archives and can be supplied upon reasonable request.

## Supplementary Materials

The following supporting information can be downloaded at: https://www.mdpi.com/article/10.3390/biom16081111/s1. Figure S1: Gating strategy for the evaluation of: lymphocyte viability, T-cell populations and frequency of IL2 ^+^ CD8^+^ T under different stimulus conditions (negative control, positive control, and Peptivator) in a PBMC sample from an rs11322783TT/TT patient; Table S1: Association of *INFG rs2830561A/T* SNP genotypes with increased percentage (<median or ≥median) of IFNγ-positive CD4^+^ or CD8^+^ T cells after stimulation with SARS-CoV-2 virus peptides; Table S2: Association of *TNFA rs1800629G/A* SNP genotypes with increased percentage (<median or ≥median) of TNFα-positive CD4^+^ or CD8^+^ T-cell subsets after stimulation with SARS-CoV-2 virus peptides; Table S3: Association of *IL2 rs2069762A/C* SNP genotypes with increased percentage (<median or ≥median) of IL-2-positive CD4^+^ or CD8^+^ T-cell subsets after stimulation with SARS-CoV-2 virus peptides; Table S4: Association of *IFNλ3* and *IFNλ4* SNP genotypes with increased percentage (<median or ≥median) of IFNγ-positive CD4^+^ T-cell subset after stimulation with SARS-CoV-2 virus peptides; Table S5: Association of *IFNL3* and *IFNL4* SNP genotypes with increased percentage (<median or ≥median) of IL-2-positive CD4^+^ T-cell subset after stimulation with SARS-CoV-2 virus peptides; Table S6: Association of *IFNλ3* and *IFNλ4* SNP genotypes with increased percentage (<median or ≥median) of IFNγ-positive CD8^+^ T-cell subset after stimulation with SARS-CoV-2 virus peptides; Table S7: Association of *IFNL3* and *IFNL4* SNP genotypes with increased percentage (<median or ≥median) of TNFα-positive CD8^+^ T-cell subset after stimulation with SARS-CoV-2 virus peptides. Table S8: Association of *IFNL4 SNP* genotypes with increased percentage (<median or ≥median) of IL-2-positive CD8^+^ T-cell subset after stimulation with SARS-CoV-2 virus peptides. Adjusted *p*-values by Bonferroni, Holms, Hochberg, and Hommel corrections.
