# Supplementary material for: IFNλ4 Gene Variants Are Associated with CD8+ T Cell IL-2 Response After COVID-19 Vaccination: A Preliminary Study
Source: Biomolecules. 2026 Jul 29;16(8):1111. doi: 10.3390/biom16081111 (PMC13511631; doi:10.3390/biom16081111)
Supplement: Supplementary file 1 [file biomolecules-16-01111-s001.zip › biomolecules-4390946-supplementary.pdf]

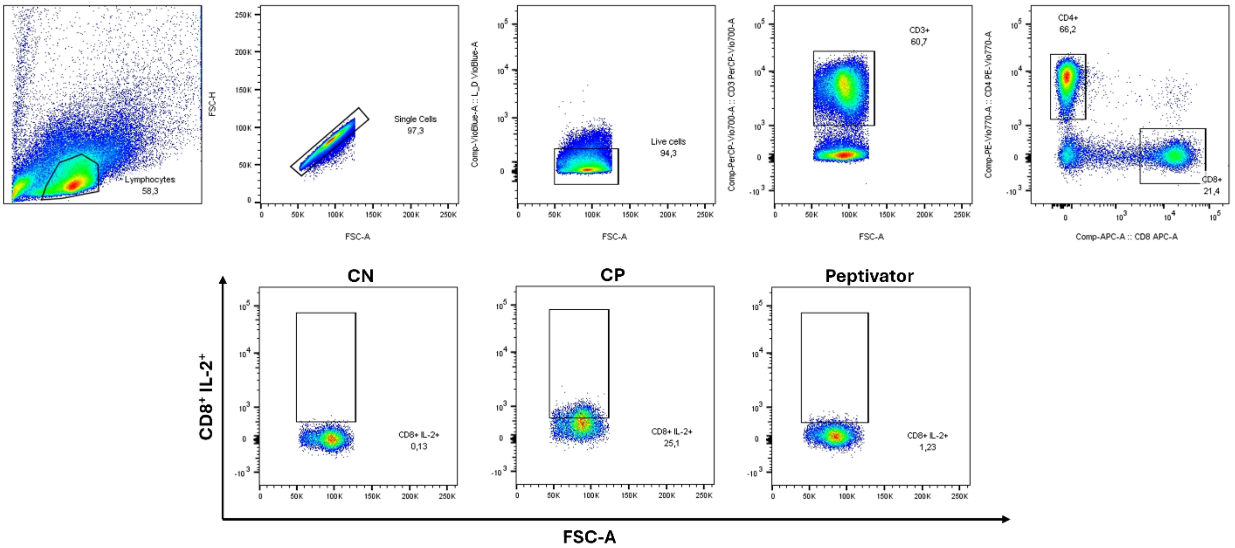

Figure S1. Gating strategy for the evaluation of: lymphocyte viability, T cell populations and frequency of IL2 + CD8+ T upon different stimuli conditions (negative control, positive control, Peptivator) in a PBMC sample from rs11322783TT/TT patient.

Table S1. Association of *INFG rs2830561A/T* SNP genotypes with the increased percentage (< Median or ≥Median) of IFNγ-positive CD4+ or CD8+ T cells after stimulation with SARS-CoV-2 virus peptides

T cell subsets			*INFG rs2830561A/T*		
			A/A	A/T	T/T
		Nr	Nr (%)	Nr (%)	Nr (%)
IFNγ positive CD4+ cells	< Median	28	10 (35.7)	16 (57.2)	2 (7.1)
	≥Median	22	5 (22.7)	16 (72.7)	1 (4.6)
	OR (95% CI)		1.89 (0.53-6.67)	0.50 (0.15-1.66)	1.61 (0.14-19-1)
	p		0.367	0.374	1.000
IFNγ positive CD8+ cells	< Median	24	10 (41.6)	13 (54.2)	1 (4.2)
	≥Median	26	5 (19.2)	19 (73.1)	2 (7.7)
	OR (95% CI)		3.00 (0.84-10.7)	0.43 (0.13-1.42)	0.52 (0.04-6.16)
	p		0.124	0.239	1.000

Table S2. Association of *TNFA rs1800629G/A* SNP genotypes with the increased percentage (< Median or ≥Median) of TNFα-positive CD4+ or CD8+ T cell subsets after stimulation with SARS-CoV-2 virus peptides

T cell subsets			*TNFA rs1800629G/A*		
			G/G	G/A	A/A
		Nr.	Nr (%)	Nr (%)	Nr (%)
TNFα positive CD4+ cells	< Median	27	17 (62.9)	8 (29.6)	2 (7.4)
	≥Median	23	16 (69.6)	5 (21.7)	2 (8.7)
	OR (95% CI)		0.74 (0.23-2.43)	1.52 (0.42-5.51)	0.84 (0.11-6.49)
	p		0.767	0.747	1.000
TNFα positive CD8+ cells	< Median	25	16 (64.0)	7 (28.0)	2 (8.0)
	≥Median	25	17 (68.0)	6 (24.0)	2 (8.0)
	OR (95% CI)		0.84 (0.26-2.70)	1.23 (0.35-4.37)	1.00 (0.13-7.72)
	p		1.000	1.000	1.000

Table S3. Association of *IL2 rs2069762A/C* SNP with the increased percentage (< Median or ≥Median) of IL-2-positive CD4+ or CD8+ T cell subsets after stimulation with SARS-CoV-2 virus peptides

T cell subsets			*IL2 rs2069762A/C*		
			A/A	A/C	C/C
		Nr.	Nr (%)	Nr (%)	Nr (%)
IL-2 positive CD4 cells	< Median	24	10 (41.7)	12 (50.0)	2 (8.3)
	≥Median	26	11 (42.3)	11 (42.3)	4 (15.4)
	OR (95% CI)		0.97 (0.32-2.99)	1.36 (0.45-4.16)	0.50 (0.08-3.02)
	p			0.777	0.669
IL-2 positive CD8+ cells	< Median	25	12 (48.0)	10 (40.0)	3 (12.0)
	≥Median	25	9 (36.0)	13 (52.0)	3 (12.0)
	OR (95% CI)		1.64 (0.53-5.09)	0.62 (0.20-1.89)	1.00 (0.18-5.51)
	p		0.567	0.571	1.000

Table S4. Association of IFNλ3 and IFNλ4 SNP genotypes with the increased percentage (< Median or ≥Median) of IFNγ-positive CD4+ T cell subset after stimulation with SARS-CoV-2 virus peptides

SNP	Genotypes	< Median Nr. (%)	≥Median Nr. (%)	OR (95% CI)	p
		28	22		
IFNL3 rs12980275A/G	A/A	7 (25.0)	2 (9.1)	3.33 (0.62-18.0)	0.266
	A/G	17 (60.7)	17 (77.3)	0.46 (0.13-1.59)	0.240
	G/G	4 (14.3)	3 (13.6)	1.06 (0.21-5.30)	1.000
IFNL3 rs8099917G/T	T/T	6 (21.4)	4 (18.2)	1.23 (0.30-5.03)	1.000
	G/T	20 (71.4)	17 (77.3)	0.74 (0.20-2.68)	0.751
	G/G	2 (7.2)	1 (4.5)	1.62 (0.14-19.1)	1.000
IFNL4 rs11322783ΔG/TT	TT/TT	15 (53.6)	14 (63.6)	0.66 (0.21-2.07)	0.569
	ΔG/TT	12 (42.8)	6 (27.3)	2.00 (0.60-6.64)	0.374
	ΔG/ΔG	1 (3.6)	2 (9.1)	0.37 (0.03-4.38)	0.576
IFNL4 rs12979860C/T	C/C	6 (21.4)	4 (18.2)	1.23 (0.30-5.03)	1.000
	C/T	19 (67.9)	15 (66.7)	0.99 (0.30-3.26)	1.000
	T/T	3 (10.7)	3 (11.1)	0.76 (0.14-4.19)	1.000

Table S5. Association of *IFNL*3 and *IFNL*4 SNP genotypes with the increased percentage (< Median or ≥Median) of IL-2-positive CD4+ T cell subset after stimulation with SARS-CoV-2 virus peptides

SNP	Genotype	< Median Nr. (%)	≥Median Nr. (%)	OR (95% CI)	p
		22	28		
IFNL3 rs12980275A/G	A/A	4 (18.2)	5 (17.85)	1.02 (0.24-4.37)	1.000
	A/G	16 (72.7)	18 (64.3)	1.48 (0.44-4.99)	0.559
	G/G	2 (9.1)	5 (17.85)	0.46 (0.08-2.64)	0.444
IFNL3 rs8099917G/T	T/T	3 (13.6)	7 (25.0)	0.47 (0.11-2.10)	0.479
	G/T	18 (81.8)	19 (67.9)	2.13 (0.56-8.16)	0.339
	G/G	1 (4.6)	2 (7.1)	0.62 (0.05-7.31)	1.000
IFNL4 rs11322783ΔG/TT	TT/TT	10 (45.45)	19 (67.8)	0.39 (0.12-1.25)	0.152
	ΔG/TT	10 (45.45)	8 (28.6)	2.08 (0.64-6.74)	0.249
	ΔG/ΔG	2 (9.1)	1 (3.6)	2.70 (0.23-31.9)	0.576
IFNL4 rs12979860C/T	C/C	4 (18.2)	6 (21.4)	0.82 (0.20-3.34)	1.000
	C/T	17 ()	17 (60.7)	2.20 (0.63-7.70)	0.240
	T/T	1 ()	5 (17.9)	0.22 (0.02-2.03)	0.211

Table S6. Association of *IFNλ3* and *IFNλ4* SNP genotypes with the increased percentage (< Median or ≥Median) of IFNγ-positive CD8+ T cell subset after stimulation with SARS-CoV-2 virus peptides

SNP	Genotype	< Median Nr. (%)	≥Median Nr. (%)	OR (95% CI)	P
		32	18		
IFNL3 rs12980275A/G	A/A	5 (15.6)	4 (22.2)	0.65 (0.15-2.819	0.705
	A/G	22 (68.8)	12 (66.7)	1.10 (0.32-3.77)	1.000
	G/G	5 (15.6)	2 (11.1)	1.48 (0.26-8.55)	1.000
IFNL3 rs8099917G/T	T/T	7 (21.9)	3 (16.7)	1.40 (0.31-6.25)	0.730
	G/T	24 (75)	13 (72.2)	1.15 (0.31-4.26)	1.000
	G/G	1 (3.1)	2 (11.1)	0.26 (0.02-3.07)	0.291
IFNL4 rs11322783ΔG/TT	TT/TT	20 (62.5)	9 (50)	1.67 (0.52-5.37)	0.551
	ΔG/TT	9 (28.1)	9 (50)	0.39 (0.12-1.30)	0.139
	ΔG/ΔG	3 (9.4)	---	---	---
IFNL4 rs12979860C/T	C/C	8 (25)	2 (11.1)	2.67 (0.50-14.2)	0.295
	C/T	20 (62.5)	14 (77.8)	0.48 (0.13-1.79)	0.351
	T/T	4 (12.5)	2 (11.1)	1.14 (0.19-6.95)	1.000

Table S7. Association of *IFNL3* and *IFNL4* SNP genotypes with the increased percentage (< Median or ≥Median) of TNFα-positive CD8+ T cell subset after stimulation with SARS-CoV-2 virus peptides

SNP	Genotype	< Median Nr. (%)	≥Median Nr. (%)	OR (95% CI)	p
		25	25		
IFNL3 rs12980275A/G	A/A	2 (8)	7 (28)	0.23 (0.04-1.21)	0.138
	A/G	19 (76)	15 (60)	2.11 (0.62-7.14)	0.364
	G/G	4 (16)	3 (12)	1.40 (0.29-7.01)	1.000
IFNL3 rs8099917G/T	T/T	5 (20)	5 (20)	1.00 (0.25-4.00)	1.000
	G/T	18 (72)	19 (76)	0.81 (0.23-2.88)	1.000
	G/G	2 (8)	1 (4)	2.09 (0.18-24.6)	1.000
IFNL4 rs11322783ΔG/TT	TT/TT	17 (68)	12 (48)	2.30 (0.73-7.27)	0.252
	ΔG/TT	7 (28)	11 (44)	0.49 (0.15-1.61)	0.377
	ΔG/ΔG	1 (4)	2 (8)	0.48 (0.04-5.66)	1.000
IFNL4 rs12979860C/T	C/C	6 (24)	4 (16)	1.66 (0.41-6.79)	0.725
	C/T	16 (64)	18 (72)	0.69 (0.21-2.29)	0.762
	T/T	3 (12)	3 (12)	1.00 (0.18-5.51)	1.000

Table S8. Association of IFNL4 SNP genotypes with the increased percentage (< Median or ≥Median) of IL-2-positive CD8+ T cell subset after stimulation with SARS-CoV-2 virus peptides. Adjusted p values by Bonferroni, Holms, Hochberg, and Hommel corrections.

SNP	raw p	Bonferroni	Holm	Hochberg	Hommel
IFNL4 rs11322783ΔG/TT	0.007	0.042	0.042	0.042	0.042
IFNL4 rs11322783TT/TT	0.021	0.126	0.105	0.105	0.105
IFNL4 rs12979860C/C	0.171	1	0.684	0.567	0.513
IFNL4 rs12979860T/T	0.189	1	0.684	0.567	0.567
IFNL4 rs12979860C/T	0.762	1	1	1	1
IFNL4 rs11322783ΔG/ΔG	1	1	1	1	1
